# Evaluating the Effect of Video Source and Other Video Characteristics on the Quality, Reliability, Actionability, and Understandability of Videos on Acromioclavicular Joint Repair

**DOI:** 10.7759/cureus.78518

**Published:** 2025-02-04

**Authors:** Abdullah B Chandasir, Justin T Skariah, Justin D Abes, Akshar Patel, Mitchell J Lomis, Noora S Chandasir, Brett D Owens, Stephen A Parada

**Affiliations:** 1 Orthopedic Surgery, Augusta University Medical College of Georgia, Augusta, USA; 2 Orthopedic Surgery, Philadelphia College of Osteopathic Medicine, Suwanee, USA; 3 Orthopedic Surgery, Brown University, Providence, USA

**Keywords:** acromioclavicular separation, patient education, sprain, student education, youtube™

## Abstract

Purpose: This study aims to evaluate video quality, reliability, actionability, and understandability differences based on length, popularity, and source credentials (physician versus non-physician). The hypothesis suggests that current videos are of low quality and limited usefulness to patients, highlighting significant disparities based on the credentials of the video source.

Methods: The phrase "acromioclavicular joint separation" was searched on YouTube. The first 100 videos that populated were selected. Of those 100, 45 were excluded based on pre-existing criteria. Two reviewers watched and graded the included videos using four established, additive algorithmic grading scales. Grades for all included videos were analyzed using R software version 4.2.3.

Results: The mean Journal of the American Medical Association (JAMA) score was 2.32 (standard deviation (SD) = 0.74), with patient-made videos having a significantly lower reliability score (p = 0.008). The mean Patient Education Materials Assessment Tool (PEMAT) understandability and actionability scores were 59.78% (SD = 15.28%) and 67.55% (SD = 15.28%) respectively. PEMAT actionability scores were positively correlated to views (p = 0.002). The average DISCERN score was 2.51 (SD = 0.70); longer videos were correlated with higher DISCERN scores (p = 0.047).

Conclusion: Analysis indicated that there were significant differences in reliability and understandability between video source types. Additionally, there was no correlation between quality and/or reliability and views, indicating that the YouTube algorithm is not an effective indicator of the quality of videos.

## Introduction

Acromioclavicular (AC) joint separations are very common in the United States, particularly in young athletes and men, and account for about 9% of all shoulder girdle injuries. These injuries are reported to be five times more common in men than in women, along with 43.5% of AC joint separations affecting adults around 20 years of age [1]. AC joint separations occur in many sports such as hockey where it is noted to be the third most commonly sustained injury [2]. In addition, a study on Major League Baseball players found that AC joint separations specifically resulted in an average of three weeks of lost game time [2], indicating that AC joint separations can have an effect on athlete livelihood and career earnings. In the digital age, online medical resources are becoming increasingly useful as a supplement to in-clinic appointments and discussions, especially when dealing with a younger patient population, as in the case of patients with AC joint separations.

YouTube is a widely used online platform with over 2.7 billion monthly active users globally. It is accessed daily by more than 122 million users who collectively stream over one billion hours of content [3]. Beyond entertainment, YouTube serves educational purposes, including healthcare and medicine. It plays a crucial role in the healthcare sector by facilitating the dissemination of information and education. Healthcare professionals use YouTube to explain medical conditions, treatments, and prevention strategies, empowering patients to make informed health decisions. The platform also hosts instructional videos on medical procedures, surgeries, and techniques, benefiting medical students, residents, and practitioners seeking to enhance their skills. The COVID-19 pandemic has further accelerated the growth of medical content on YouTube and other online platforms [4].

Nevertheless, it is crucial to highlight the absence of regulatory oversight in the realm of healthcare content on YouTube, leaving the legitimacy of information delivered by sources uncertain. Recognizing this concern, numerous recent studies have been conducted to assess the reliability of healthcare content on YouTube as a credible source of information. These studies examining various medical conditions have consistently concluded that the quality and reliability of medical content on YouTube are often poor in quality and reliability [5,6]. This study aims to assess the quality, readability, and absorbability of content on YouTube regarding acromioclavicular joint separations. The analysis of this study is highly relevant in orthopedic medicine due to the nature of AC joint separations and their relatively high rate of occurrence, particularly in athletes of contact sports such as rugby, wrestling, hockey, football, and baseball [7]. Notably, there remains a lack of YouTube content analysis regarding AC joint separations, making this study increasingly relevant as it pertains to a rather prevalent injury in sports medicine. The purpose of this study is to determine if there are any discrepancies in video quality, reliability, actionability, and understandability based on length, popularity, and source credentials (physician versus non-physician healthcare representative). The hypothesis of this study maintains that YouTube videos regarding AC joint separations available to patients are of low quality and limited usefulness, with there being significant quality differences based on the credentials of the video source.

## Materials and methods

YouTube study design

Two video reviewers were recruited through interest groups via a non-blinded search to analyze videos on AC joint separation. The latest version of Google Chrome was used as the preferred browser for video selection and review. The reviewers used private browsing via incognito mode to prevent video bias based on YouTube search algorithms. All videos were selected from www.YouTube.com. Both reviewers performed a video search using the following phrase in the YouTube search bar: "acromioclavicular joint separation". Reviewers selected the first 100 videos on YouTube and verified that the video search was identical.

Video inclusion and exclusion criteria

**Figure 1:**
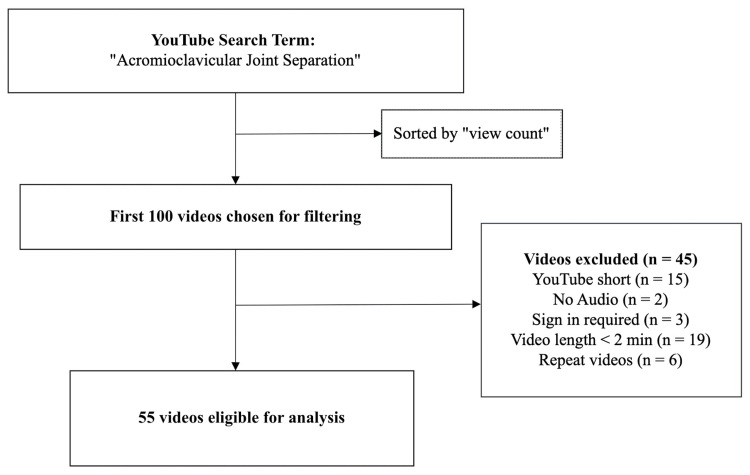
Search Methodology for YouTube Videos Pertaining to Acromioclavicular Joint Separation

Reviewers only selected videos that included information involving AC joint separations. Videos were filtered by the number of views, and the first 100 videos were selected in order. From these selected videos, certain videos were excluded secondary to exclusion criteria. The exclusion criteria were selected based on similar studies that reviewed the educational relevance of YouTube videos for medical procedures and analysis. The exclusion criteria were as follows: (1) no audio, (2) YouTube short video, (3) sign-in required, (4) video length is less than two minutes, and (5) repeat of a previously included video. Out of the 100 chosen videos, 55 were included for analysis, while 45 were excluded based on the above criteria (Figure 1).

General video information

For each video analyzed, general video information was recorded for further review. This information included the following: (1) video title, (2) author, (3) date uploaded, (4) days since upload, (5) video duration (in seconds), (6) total views, (7) views ratio (total views / days since upload), (8) total likes, (9) the number of comments, (10) uploader/source (physician, device company, non-physician health professional, patient), (11) number of subscribers, (12) YouTube verification, (13) intended audience, and (14) medical disclaimer included.

Video data collection and source demographics

The views ratio was calculated by dividing the total video views by the days since the video was uploaded (views ratio = total views / days since upload). To determine the uploader source, the following categories were used: (1) physician (this category included a medical doctor, a doctor of osteopathic medicine, or a doctor of podiatric medicine), (2) non-physician health professional (this includes chiropractors, physical therapists, physiotherapists, nurse-practitioners, occupational therapists, nurses, or nurse practitioners), (3) academic source (this included a research institution, a school, or a university), (4) patient (who previously experienced an AC joint injury), or (5) device company. YouTube verification was determined by the presence of a verification checkmark next to the uploader's YouTube handle under their account. Information was also collected on the intended audience if this was specified in the video itself or if it was included in the video description (underneath the video). The use of a medical disclaimer was accessed based on whether the video had any warning regarding the intended use and adherence to the medical information provided in the video. This disclaimer could be present in the video itself or in the video description.

Journal of American Medical Association (JAMA) grade calculation

The Journal of American Medical Association (JAMA) benchmark criteria was used to assess the reliability of the analyzed YouTube videos [5]. The JAMA criteria offer a comprehensive framework for evaluating the reliability and accuracy of video content. The four criteria used in the JAMA benchmark are authorship, attribution, disclosure, and currency [5]. These criteria consist of four distinct factors, with each criterion being assigned a score of one point, culminating in a maximum total score of four points (Table 1). A higher score indicates a heightened level of reliability and accuracy in the video content, while a score of zero indicates a diminished level of reliability and accuracy [8]. A cutoff score of three or more was used to indicate a "good" JAMA score, in accordance with established guidelines from current literature [4].

**Table 1:** JAMA Benchmark Criteria JAMA: Journal of American Medical Association

Criteria	Description
Authorship	Authors and contributors of the video must be provided, along with their credentials.
Attribution	References and sources for all content should be listed clearly, and all relevant copyright information noted.
Disclosure	Website "ownership" should be prominently and fully disclosed, as should any sponsorship, advertising, underwriting, and commercial funding.
Currency	Dates that content was posted and updated should be indicated.

Patient Education Materials Assessment Tool for Audiovisual Materials (PEMAT-A/V) understandability and actionability score calculation

The assessment of individual videos' understandability and actionability was performed using the Patient Education Materials Assessment Tool for Audiovisual Materials (PEMAT-A/V) [5]. This tool was specifically selected for its ability to evaluate various aspects such as content, word choice and style, organization, layout and design, and the use of visual aids, which are encompassed within the understandability section comprising 13 items (Table 2). The second section focuses on actionability and consists of four items (Table 3). Each item is assigned a score of zero ("disagree"), one ("agree"), or N/A ("not applicable"). Subsequently, the percentage scores for both sections are calculated by dividing the achieved points by the total number of items evaluated for each section. PEMAT-A/V scores range from 0% to 100%, where higher values generally indicate a higher level of understandability or actionability [9]. Scores of 70% or higher were used to classify videos as having good actionability and understandability [4].

**Table 2:** PEMAT Understandability Grading Scale PEMAT: Patient Education Materials Assessment Tool

Item number	Item	Grading option
1	The material makes its purpose completely evident.	Disagree = 0, agree = 1
2	The material uses common, everyday language.	Disagree = 0, agree = 1
3	Medical terms are used only to familiarize the audience with the terms. When used, medical terms are defined.	Disagree = 0, agree = 1
4	The material uses the active voice.	Disagree = 0, agree = 1
5	The material breaks or "chunks" information into short sections.	Disagree = 0, agree = 1
6	The material's sections have informative headers.	Disagree = 0, agree = 1
7	The material presents information in a logical sequence.	Disagree = 0, agree = 1
8	The material provides a summary.	Disagree = 0, agree = 1
9	The material uses visual cues (e.g., arrows, boxes, bullets, bold, larger font, highlighting) to draw attention to key points.	Disagree = 0, agree = 1
10	The material uses visual cues (e.g., arrows, boxes, bullets, bold, larger font, highlighting) to draw attention to key points.	Disagree = 0, agree = 1, no text/all text is narrated = NA
11	The material allows the user to hear the words clearly (e.g., not too fast, not garbled).	Disagree = 0, agree = 1, no narration = NA
12	The material uses illustrations and photographs that are clear and uncluttered.	Disagree = 0, agree = 1, no visual aids = NA
13	The material uses simple tables with short and clear row and column headings.	Disagree = 0, agree = 1, no tables = NA

**Table 3:** PEMAT Actionability Score Grading Criteria PEMAT: Patient Education Materials Assessment Tool

Item number	Item	Grading option
1	The material clearly identifies at least one action the user can take.	Disagree = 0, agree = 1
2	The material addresses the user directly when describing actions.	Disagree = 0, agree = 1
3	The material breaks down any action into manageable, explicit steps.	Disagree = 0, agree = 1
4	The material breaks down any action into manageable, explicit steps.	Disagree = 0, agree = 1, no charts, graphs, tables, diagrams = NA

Modified DISCERN calculation

The Modified DISCERN tool is used to measure the quality and reliability of the video [10]. It is calculated based on five criteria, with one point assigned for each criterion present in the video. There is a maximum of five points available for this scale (Table 4). Higher DISCERN values for a particular video are associated with higher reliability for the content in the video [11]. A cutoff of four was used to classify videos as having a high DISCERN score [4].

**Table 4:** Modified DISCERN Grading Criteria

Item number	Item	Grading Option
1	Are the aims clear and achieved?	Disagree = 0, agree = 1
2	Are reliable sources of information used?	Disagree = 0, agree = 1
3	Is the information presented balanced and unbiased?	Disagree = 0, agree = 1
4	Are additional sources of information listed for patient reference?	Disagree = 0, agree = 1
5	Are areas of uncertainty mentioned?	Disagree = 0, agree = 1

Video bias

Scoring bias was addressed by two separate reviewers to calculate the scores for each video. The potential for video selection bias was addressed by using a private browser when performing the YouTube search to remove algorithm changes to search data.

Statistical analysis

R version 4.2.3 (Shortstop Beagle) was used to calculate descriptive statistics as well as for statistical analysis. To analyze and confirm inter-rater reliability, Cronbach's alpha coefficient was assessed for each rating scale. Kruskal-Wallis tests and Mann-Whitney U tests were performed when comparing categorical variables; categories were assigned dummy variables. Pearson's correlation tests were used when comparing numerical variables with respective scores.

## Results

Descriptive data and video statistics

**Figure 2:**
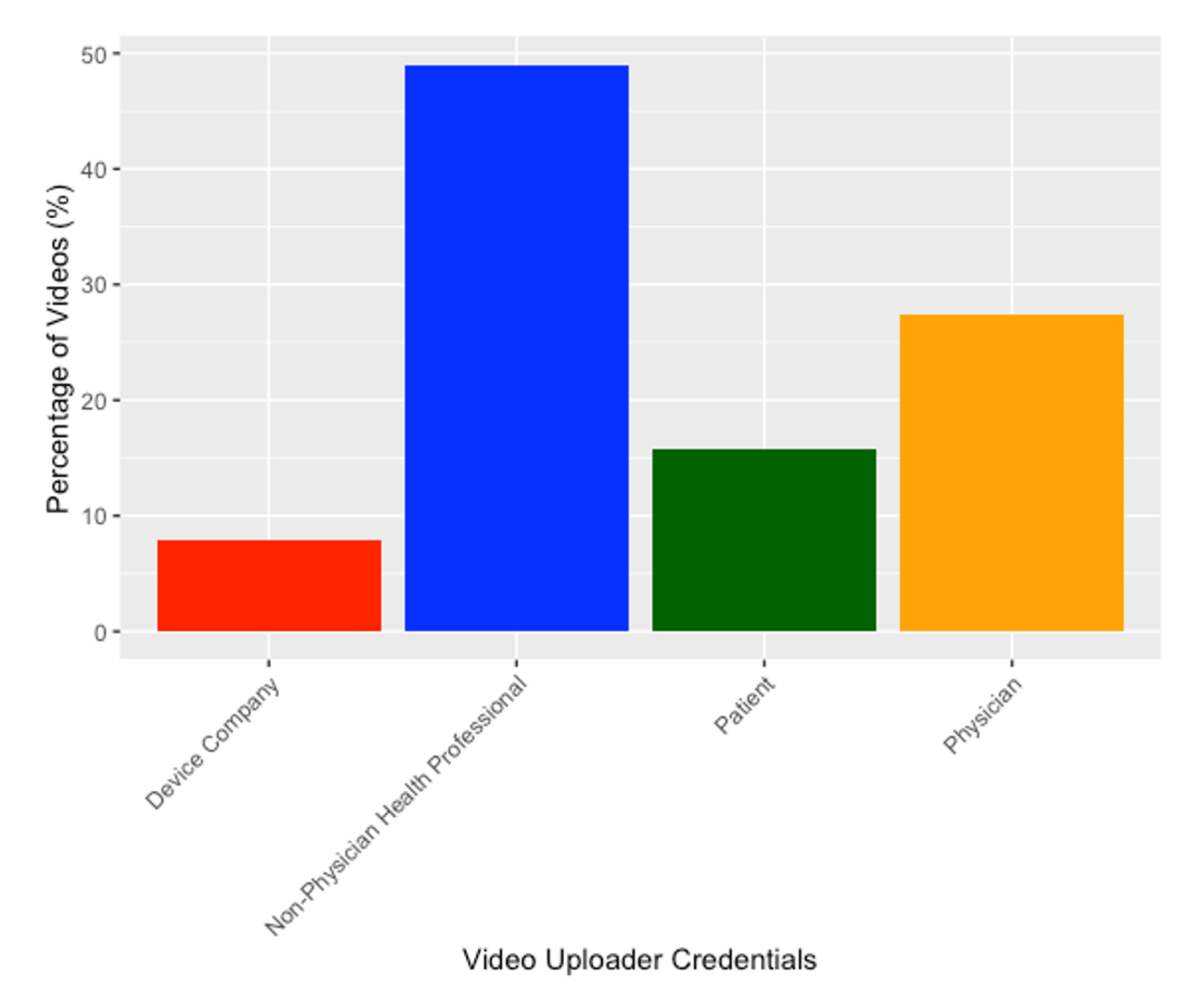
Distribution of Videos by Uploader/Source Classification

Videos had a mean duration of 7.98 ± 6.30 minutes, with a range of 2.12-36.35 minutes. The mean number of views, likes, and comments for all videos was 132,274.2 (SD = 193809), 193,809 (SD = 2,707.597), and 147.86 (SD = 214.14), respectively. Four videos had disabled comment sections. Videos received an average of 75.25 views per day (SD = 92.83). The average number of days that the videos analyzed had been on YouTube was 1,853.17 days (SD = 1,369.46) or about five years. Among the videos that were analyzed, non-physician health professionals produced the most videos (n = 25, 46.30%), followed by physicians (n = 15, 27.78%) (Figure 2). The mean number of subscribers that each creator had was 591,231.9 (SD = 1,305,764). The majority of videos did not have an intended audience (n = 37, 68.52%). All videos that had a specified audience were targeted toward patients (n = 17, 31.48%). There were 39 authors who were represented in the videos that were chosen for analysis. Six (15.38%) authors had more than one video that was part of the sample being analyzed. The authors "Nabil Ebrahim", "Bob and Brad", and "Ortho Films" all had four (7.41%) videos as part of the chosen sample.

JAMA score

**Figure 3:**
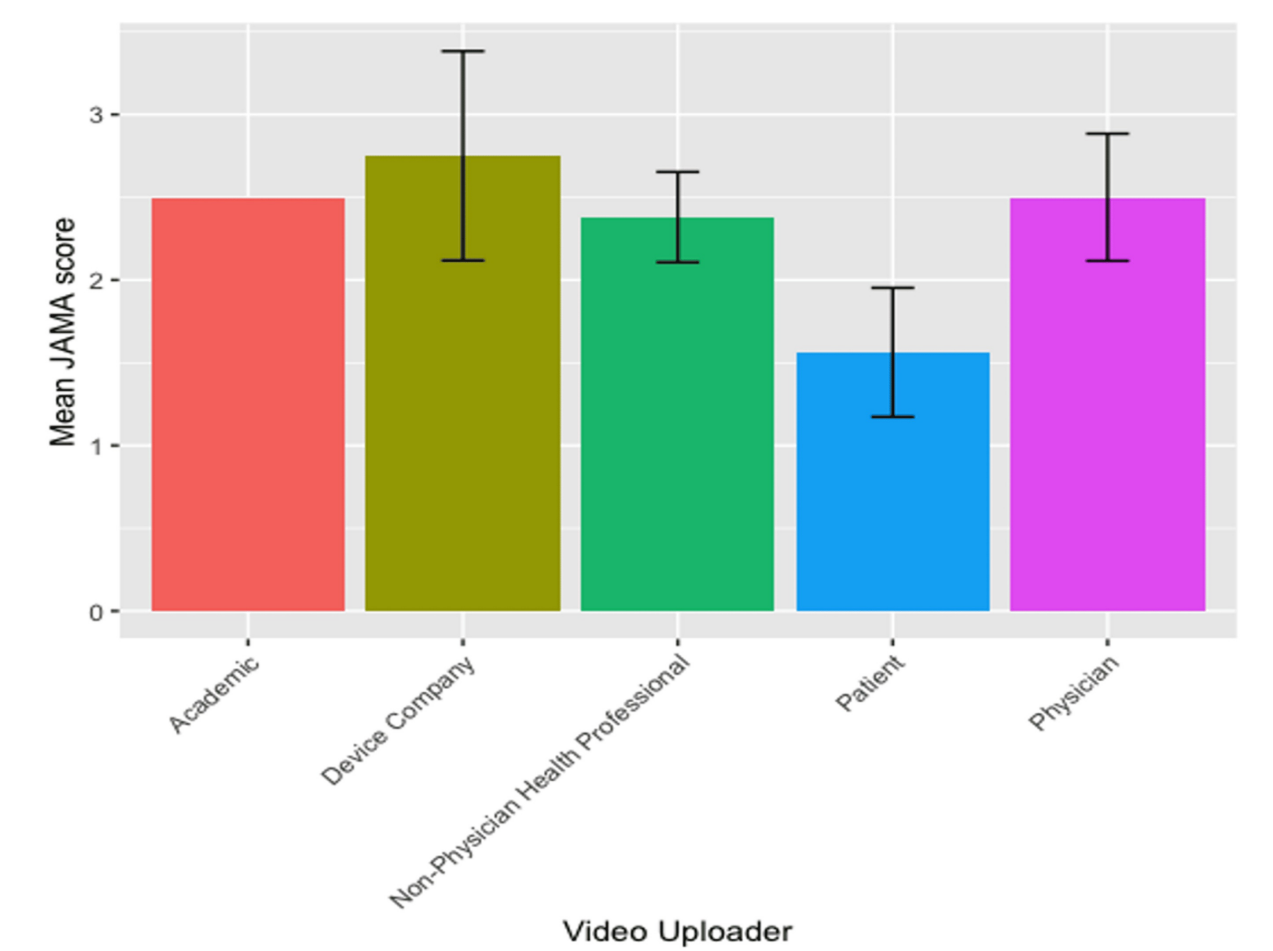
Mean JAMA Benchmark Score by Uploader Classification JAMA: Journal of American Medical Association

The average JAMA score for analyzed videos was 2.32 (SD = 0.74). A majority of videos had a mean JAMA grade of three out of four (n = 14, 26.9%), followed by two out of four (n = 11, 21.2%). Only four (7.7%) videos had a mean JAMA grade higher than three, indicating that they were of good reliability. Many of the videos lost points due to the attribution criteria (references, sources, and copyright information listed for each video). Most videos gained points due to the currency criteria (video upload date listed). There was a statistically significant decrease in the JAMA scores of videos made by patients (1.6) as compared to videos made by physicians (2.5) and non-physician healthcare providers (2.4) (p = 0.008) (Figure 3). There was no correlation between the JAMA score and views, views per day, likes, or comments. There was also no correlation found between the JAMA score and the intended audience, subscriber amount, YouTube verification status, or medical disclaimer status (Table 5).

**Table 5:** Comparison of Mean JAMA Score by Different Video Characteristics The test statistics provided are the H statistic for the categorical variables (video source, intended audience, verification status, and medical disclaimer) and the t-score for numerical values (views, views ratio, likes, comments, and subscriber amount). * indicates a statistically significant difference. JAMA: Journal of American Medical Association

Video characteristic	P-value	Test statistic value
Video source	0.008*	15.63
Views	0.07	17284
Views ratio	0.12	18280
Likes	0.54	20138
Comments	0.38	17324
Intended audience	0.70	2.33
Subscriber amount	0.38	20526
Verification status	0.66	6.27
Medical disclaimer	0.15	4.46

PEMAT score

**Figure 4:**
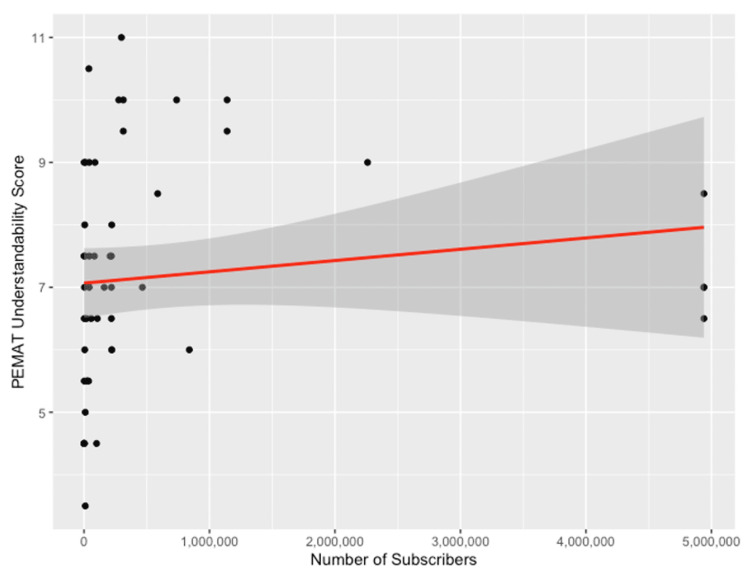
Correlation Between the Number of Subscribers and Mean PEMAT Understandability Score PEMAT: Patient Education Materials Assessment Tool

The average PEMAT understandability score for the analyzed videos was 59.78% (SD = 15.28%). There were 23 videos that had a score higher than 70%, indicating that they were of good understandability. Many videos lost points due to PEMAT criteria #8 for video summaries (the video provides a summary of the information provided) as well as #13 with the use of simple tables. Most videos gained points due to criteria #2 for the use of everyday language and #4 for using active voice in the videos. There was a significant positive correlation between the number of subscribers and the average PEMAT understandability score (p = 0.048) (Figure 4). Additionally, there was a significant difference in the average PEMAT understandability scores based on video uploader/source, with videos made by patients (5.06) having lower average understandability scores than non-physician healthcare professionals (7.20) (p = 0.002) and physicians (p < 0.001). Additionally, videos made by non-physician healthcare professionals (7.20) had a lower average understandability score than videos made by physicians (8.32) (p = 0.049). There was also a significant difference between the average understandability scores of videos that contained a medical disclaimer; videos without a medical disclaimer (6.78) had a lower mean understandability score than those that contained a medical disclaimer (8.12) (p = 0.01). There was no correlation between PEMAT understandability scores and views, views ratio, days since upload, likes, comments, YouTube verification status, and intended audience (Table 6).

**Table 6:** Comparison of Mean PEMAT Understandability Score by Different Video Characteristics The test statistics provided are the H statistic for the categorical variables (video source, intended audience, verification status, and medical disclaimer) and the t-score for numerical values (views, views ratio, likes, comments, and subscriber amount). * indicates a statistically significant difference. PEMAT: Patient Education Materials Assessment Tool

Video characteristic	P-value	Test statistic
Video source	<0.001*	36.12
Views	0.43	20056
Views ratio	0.16	18353
Likes	0.52	17573
Comments	0.25	19142
Intended audience	0.15	13.50
Subscriber amount	0.048*	12377
Verification status	0.62	9.45
Medical disclaimer	0.01*	29.14

**Figure 5:**
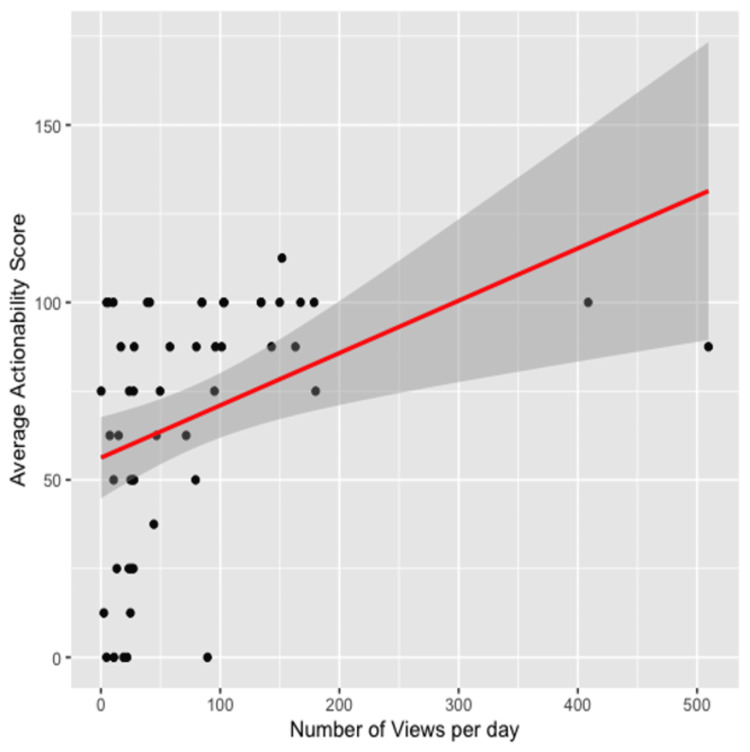
Correlation Between the Number of Views Per Day and PEMAT Actionability Score PEMAT: Patient Education Materials Assessment Tool

The average PEMAT actionability score was 67.55% (SD = 34.4%). There was a statistically significant positive correlation between the number of views a video had received and the average PEMAT actionability score (p = 0.002, r = 0.33). The same trend was found when comparing the views ratio and the actionability score (p < 0.001, r = 0.40) (Figure 5). In addition, videos made with patients as the intended audience had a higher actionability score (p = 0.01). There was no other correlation found between the actionability score and days since video upload, video duration, likes, comments, video uploader, subscribers, verification status, and medical disclaimer status (Table 7).

**Table 7:** Comparison of Mean PEMAT Actionability Score by Different Video Characteristics The test statistics provided are the H statistic for the categorical variables (video source, intended audience, verification status, and medical disclaimer) and the t-score for numerical values (views, views ratio, likes, comments, and subscriber amount). * indicates a statistically significant difference. PEMAT: Patient Education Materials Assessment Tool

Video characteristic	P-value	Test statistic
Video source	0.08	12.15
Views	0.002*	13734
Views ratio	<0.001*	11329
Likes	0.52	11594
Comments	0.14	16124
Intended audience	0.76	14.95
Subscriber amount	0.18	17238
Verification status	0.35	5.01
Medical disclaimer	0.31	11.31

DISCERN quality and reliability score

The mean DISCERN score for the videos analyzed was 2.51 (SD = 0.70). Most videos lost points due to the videos missing valid sources and additional sources not being specified. Most videos gained points due to the video being clear, short, and understandable. There was a statistically significant positive correlation found between videos of a longer duration and DISCERN quality and reliability score (p = 0.047, r = 0.56). There was no other correlation found between DISCERN quality and reliability score and views, views per day, days since upload, likes, comments, video uploader, subscribers, verification status, and medical disclaimer status (Table 8).

**Table 8:** Comparison of Mean DISCERN Score by Different Video Characteristics The test statistics provided are the H statistic for the categorical variables (video source, intended audience, verification status, and medical disclaimer) and the t-score for numerical values (video length, views, views ratio, likes, comments, and subscriber amount). * indicates statistical significance.

Video characteristic	P-value	Test statistic
Video source	0.24	10.36
Video length	0.047*	25807
Views	0.14	23785
Views ratio	0.22	26647
Likes	0.52	25223
Comments	0.14	17249
Intended audience	0.33	4.33
Subscriber amount	0.15	22497
Verification status	0.72	1.28
Medical disclaimer	0.65	3.18

## Discussion

A statistically significant difference was found in the JAMA score between videos made by patients and videos made by non-physician health professionals. This difference is expected, as the JAMA score utilizes criteria aimed at analyzing academic and professional content; patient videos were mostly aimed at providing anecdotal evidence to other potential or current patients about injury, procedure, and recovery. This disparity in reliability scores reflects similar findings indicating that YouTube videos on Achilles tendon tears made by non-physician professionals were of lower quality [12]. There was no correlation found between the mean JAMA scores and views, views per day, likes, comments, or subscribers. This indicates that the YouTube algorithm does not factor in reliability and accuracy as determined by the JAMA score. This finding is similar to those in many other studies that analyzed the quality and reliability of YouTube videos pertaining to other injuries; one study found that there was no correlation between views and quality in videos on ankle fractures. The same finding was discovered in an analysis of YouTube videos about clubfoot [4]. This lack of correlation indicates that the YouTube algorithm does not populate the videos of the highest quality first. Therefore, instead of clicking on the first video that is populated from the search, it is important to take time and analyze credentials and sources of videos before watching them and recommending them to patients.

The mean PEMAT understandability score was low, indicating that most of the videos that were analyzed were difficult to understand or comprehend from a patient's perspective. Videos made by patients scored significantly lower than those made by physicians, non-physician healthcare professionals, and device companies. This can be attributed to patients' videos mainly consisting of anecdotal accounts, where they verbally shared their procedure and recovery experiences. These accounts are typically presented chronologically, relying solely on spoken narration without the incorporation of visual aids, chunking of information, and summaries. Videos made by non-physician healthcare professionals were also found to have lower understandability scores than those made by physicians. This finding is consistent with other studies that have found that videos made by physicians tend to be of higher understandability; one study found that videos made by physicians on phimosis tended to have higher understandability [13]. Videos with medical disclaimers were also found to have higher understandability scores than videos without medical disclaimers. However, this is most likely due to the fact that most videos that contained medical disclaimers were ones that were made by physicians, reflecting the previously mentioned finding. There was no correlation between PEMAT understandability scores and views, views ratio, days since upload, likes, comments, or YouTube verification status. This finding reflects the fact that the YouTube algorithm does not consider the quality of understandability when ranking videos. In addition, because of the disparities in understandability of videos between videos made by patients versus non-physician healthcare providers versus physicians, it is important to thoroughly vet medical videos before using them either as a provider or as a patient. Lower understandability can lead to misinformation, improper action, or unnecessary stress for patients. Therefore, screening of videos before recommending them to patients as supplementary information is paramount to patient satisfaction and proper treatment.

The mean PEMAT actionability score was low, suggesting that many of the videos on AC joint separation failed to provide clear, actionable steps for patients. Videos that scored higher on the PEMAT actionability score had both more views as well as more views per day. This finding is notable in that it is one of few that has found that views and views per day are correlated with higher quality in regard to actionability, indicating that videos that provide better, clearer steps of action to patients may be ranked higher by the YouTube algorithm. Previous studies have found that YouTube videos often have a wide range of PEMAT actionability scores, with some scoring highly and others scoring poorly. A previous study on the quality, reliability, and popularity of YouTube videos on urticaria found that the overall actionability score of videos on urticaria was low. In addition to that, the study also discovered discrepancies in the actionability scores of videos made by physicians versus non-physician healthcare professionals [14]. While these findings were not reflected in our study, the finding echoes previous implications for thorough filtering of videos before either personal use or for supplementary use as a physician. Videos aimed at patients were also found to have higher actionability scores. This finding was promising, as it indicates that videos that were made with the intention of educating patients provided meaningful, clear actionable steps to take. Videos that have higher actionability allow patients to take their health into their own hands and work on their well-being in tandem with physicians, which can lead to better outcomes. This can also give patients a sense of control over their own health, which can potentially lead to better outcomes and less stress over certain ailments.

The mean DISCERN score for the videos analyzed was found to be low, indicating poor quality and reliability. These findings are reflected in many previous studies that analyzed videos on many different injuries and treatments in orthopedics [3,10,15]. One study found that the average DISCERN score for videos on carpal tunnel syndrome was 33.62 on the extended DISCERN scale, indicating that videos on carpal tunnel syndrome were of low quality and reliability [10]. Similar results were found in studies that graded videos on lumber fusion and lumbar arthroscopy [15] and ulnar collateral ligament injury and management [3]. The prevalence of low-quality videos on YouTube remains a concern for both healthcare professionals and patients. Longer videos are found to have a higher DISCERN score; this finding is consistent with others evaluating YouTube videos for different conditions, which found that longer videos are often found to be of higher quality and reliability [3,14]. However, it is important to note that although this finding is common, video creators should strive to find a balance between incorporating enough information to make a high-quality resource without making the video unnecessarily long, which can cause viewers to lose interest [12].

Studies have shown that 80% of internet users seek health-related information as part of their regular usage, influencing 75% of the medical decisions of these individuals [16]. This information shows the power and influence that online platforms such as Google and YouTube can have on patients [17]. Globally, Google and YouTube are used for information, with the term "acromioclavicular joint separation" searched on Google on an average of 12,100 times per month [18]. This popular search term leads to many YouTube videos regarding AC joint separations. These videos have the potential to be used as a supplement by healthcare providers to better inform patients and educate them on their care. YouTube has become one of the most accessible online platforms for patient information over the past decades. However, there has been increasing evidence in orthopedic surgery, as well as other fields of medicine, that indicates that the quality, reliability, and understandability of videos found on YouTube are often poor in medical education [5,9]. Therefore, this study sought to determine the quality, readability, actionability, and absorbability of YouTube videos that aim to educate viewers on AC joint separations. There was no previous study that analyzed the effectiveness and quality of online videos on AC joint separation on YouTube, which often serves as a patient's first line of health education and information pertaining to the injury. Our findings supported the fact that the majority of educational videos on AC joint separation were of low quality, reliability, actionability, and understandability. The results were in line with previous studies analyzing other pathologies and the quality of educational YouTube videos pertaining to them [1,12,14,19]. A study on YouTube videos regarding COVID-19 noted that a significant number of videos on COVID-19 were posted by non-physicians, which could lead to an increase in misinformation [1]. Another study on the quality, reliability, and actionability of YouTube videos on phimosis found that a majority of videos had low scores for quality, and the overall misinformation score was high [12]. A study on urticaria videos on YouTube also found that there was a statistically significant difference between videos made by physicians and videos made by other sources, with videos made by physicians being of higher quality [14]. This study underlines the need for regulation and oversight of the YouTube algorithm, as well as the differences in video quality between video uploaders and the intended audience.

Limitations of this study may be due to data collection criteria and YouTube algorithm characteristics. Out of the many videos that appeared in the initial YouTube search, only the first 100 were considered for analysis. There could possibly be higher- or lower-quality videos that were part of the group of videos that were not included for consideration for analysis. In addition to that, the videos were found through the search of the term "acromioclavicular joint separation"; the population of videos that appear to patients could change based on the exact phrasing they use to search for videos. In addition, the heterogeneity of the videos, especially in terms of time since publication and other details in the videos, could serve as confounding factors in the analysis of the videos.

## Conclusions

The findings of the study indicate that the quality, reliability, understandability, and actionability of YouTube videos on AC joint separation are poor, and more work needs to be done by video producers to upload quality content that can better supplement healthcare provider care and patient education. In addition, there are discrepancies in video quality and reliability by provider source, signifying the importance of thorough vetting and research before the use of videos either as a supplement to provide to patients or through online searches by patients themselves. There is also no positive correlation between the quality of videos and the number of views or views per day, indicating that the YouTube algorithm does not select videos by their quality.
